# Intermittent Fasting in Cardiovascular Disorders—An Overview

**DOI:** 10.3390/nu11030673

**Published:** 2019-03-20

**Authors:** Bartosz Malinowski, Klaudia Zalewska, Anna Węsierska, Maya M. Sokołowska, Maciej Socha, Grzegorz Liczner, Katarzyna Pawlak-Osińska, Michał Wiciński

**Affiliations:** 1 Department of Pharmacology and Therapeutics, Faculty of Medicine, Collegium Medicum in Bydgoszcz, Nicolaus Copernicus University, M. Curie 9, 85-090 Bydgoszcz, Poland; klaudiazalewska95@wp.pl (K.Z.); anka.wesierska@tlen.pl (A.W.); msokolowska@trentu.ca (M.M.S.); licznergrzegorz@gmail.com (G.L.); wicinski4@wp.pl (M.W.); 2 Department of Obstetrics, Gynecology and Gynecological Oncology, Faculty of Medicine, Collegium Medicum in Bydgoszcz, Nicolaus Copernicus University, Ujejskiego 75, 85-168 Bydgoszcz, Poland; msocha@copernicus.gda.pl; 3 Department of Pathophysiology of Hearing and Balance System, Faculty of Medicine, Collegium Medicum in Bydgoszcz, Nicolaus Copernicus University, M. Curie 9, 85-090 Bydgoszcz, Poland; osinskak1@wp.pl

**Keywords:** intermittent fasting, cardiovascular diseases, lipid profile, atherosclerosis, blood pressure

## Abstract

Intermittent fasting is a form of time restricted eating (typically 16 h fasting and 8 h eating), which has gained popularity in recent years and shows promise as a possible new paradigm in the approach to weight loss and the reduction of inflammation, and has many potential long term health benefits. In this review, the authors will incorporate many aspects of fasting, mainly focusing on its effects on the cardiovascular system, involving atherosclerosis progression, benefits for diabetes mellitus type 2, lowering of blood pressure, and exploring other cardiovascular risk factors (such as lipid profile and inflammation).

## 1. Introduction

Cardiovascular diseases are a serious problem in the modern world. According to WHO (World Health Organization) data, 17.9 million people die every year due to cardiovascular diseases, which is about one third of all deaths [1]. They most often affect people over 45 years of age. The mortality rate is different in both sexes in any given period of life. Between the ages of 45–59, men predominate, while after the age of 60, the death rate is higher in women [2]. These differences are related to the cardio protective effect of estrogens in premenopausal women [3]. Modifiable and unmodifiable factors contribute to the development of cardiovascular diseases. Age, gender, or genetic determinants are factors beyond our control. However, smoking, obesity, lack of physical activity, disorders of lipid metabolism, hypertension, diabetes, and poor diet are among the modifiable factors [4]. The coexistence of two or more risk factors increases the likelihood of the disease occurrence. Treatment of cardiovascular diseases includes patient training in the context of the importance of lifestyle changes, taking into account pharmacotherapy and invasive therapy [5].

The control of risk factors allows for a reduction of mortality and pathogenicity, in particular in patients with unrecognized cardiovascular disease [6]. Lifestyle adjustments, i.e., smoking cessation, increasing physical activity, or ensuring proper body weight, reduces the risk of cardiovascular disease. With the growing problem of obesity in the world, diet changes are an important modifiable factor. Meals should be varied, similar to the Mediterranean diet. It is recommended to eat large amounts of vegetables, fruit, fish, and only whole-grain bread. The eating of red meat, sweetened beverages, and excessively salty foods (daily salt intake < 5 g) should be avoided [5,7]. Large amounts of alcohol should also be avoided. Consumption of spirits should be limited to 10 g/day in women and 20 g/day in men [5].

Along with the growing epidemic of obesity, the search for new and effective dietetic solutions aimed at reducing calories and reducing body mass was initiated. Currently, the intermittent fasting (IF) diet is gaining popularity [8]. For many people, it is considered to be less restrictive compared to traditional methods of calorie restriction (calorie restriction) [9]. It involves taking a normal, daily caloric intake with the use of short, strict calorie restriction [10]. Meals are only consumed within a strictly defined time within a day or week [8]. There are two basic varieties of the IF diet. The most popular variation is time-restricted feeding. It may be used in three variants: 16/8, 18/6 and 20/4. 16:8, consisting of a 16-h fast, and then an 8-h nutritional window. In a more rigorous approach, the nutritional window can be shortened to 4 h [8]. Another protocol consists of a 24-h fasting period, alternated with a 24-h eating period, repeated two or three times a week. There are two possible systems, 5:2 or 4:3. In the 5:2 system, in which caloric restriction is used for two days a week, and a regular diet for 5 days. The literature describes fasting periods as a consumption of about 400–600 kcal/day. Most people separate their fasting days [11]. In 2016, Carter et al. compared the effectiveness of the IF diet in the 5:2 system with the continuous energy restriction (CER) diet. The authors found that the IF diet may be an alternative for weight loss and glycemic control during 12 weeks. Moreover, the IF diet presents a useful substitute to obese/overweight patients who find the CER diet difficult to maintain [12].

The subtype of the IF diet is the ADF diet (alternate day fasting). It consists of alternating the day when the energy limit is 75%, the so-called “fast day” and “feeding day”, during which food is eaten ad libitum (at one’s pleasure, shortened to “ad lib”). The use of IF allows body weight to be reduced and is cardio protective [9]. Cardioprotective effects of the ADF diet are probably associated with a reduction of fat tissue (especially visceral fat tissue), increased adiponectin concentration, and decreased leptin and low-density lipoprotein (LDL) concentration [13]. In other studies, individuals following the ADF diet, after a period of dietary restriction, observed an increase in hunger during the day, but also increase in satiety after a meal, which resulted in consumption difficulties [14].

Time-restricted feeding (TRF) is a type of IF diet that focuses on eating within a particular window of time. Protocol may vary according to individual preferences and lifestyle. TRF involves limiting intake to several hours (6–12 h). The TRF diet is of special interest among physically active people due to reports on its effect on weight reduction while maintaining muscle mass. Thus, it may help athletes to achieve the desired body mass for a specific sport category. Moro et al. provided a trial on 34 resistance trained males who were randomly assigned to a time-restricted feeding or normal diet group. The interventional group ate 100% of their energy needs during an 8 h eating window each day with their caloric intake divided into three time-points (1 p.m.; 4 p.m.; 8 p.m.). The normal diet group consumed 100% of their energy needs divided into three time-points (8 a.m.; 1 p.m.; 8 p.m.). After 8 weeks, analysis showed a decrease in fat mass in the TRF group compared to the normal diet group, while the fat-free mass, and the muscle area of the arm and thigh remained unchanged in both groups [15].

In contrast to traditional IF, TRF is usually performed on a daily basis and does not need prescribed restrictions. Additionally, the fasting window may be planned during nighttime. Thus, it can help some individuals to avoid night eating and follow a circadian rhythm.

Table 1 presents comparison of intermittent fasting protocols.

The above-mentioned diets can be successfully used in people who want to reduce their weight to improve their health, but can also be implemented in a population of patients in whom obesity is an important risk factor for the development of type II diabetes [10]. In addition, the IF diet can be used as a supplement to training processes for people with a normal weight who want to improve their health regardless of their weight loss. In these people, intensive energy restriction (IEF) requires a concentration on energy restriction (ER) for specific days of the week, which is easier to achieve than daily, continuous energy reduction, as is the case in traditional CER (continuous energy restriction) [16]. Moreover, many beneficial metabolic effects, taking place during weight loss and energy limitation, are associated only with the limitation of energy and are suppressed when a person no longer has a negative energy balance [17].

However, according to the National Institute for Health and Care Excellence (NICE) guidelines for the treatment of obesity in adults, routine use of very low calorie diets (VLCDs) in the therapeutic regimen of obesity in adults is not recommended. According to this institute, such an approach should be recommended when there is a clinical justification for rapid weight loss and it must supply all necessary nutrients. Additionally, it should be attempted for a maximum of 12 weeks (continued continuously or intermittently) [10].

Many studies based on human and animal models on weight loss using an IF diet confirm the reduced risk of developing cardiovascular diseases. This is related to the modulating effect of the IF diet on various risk factors of development, such as obesity, improper diet, insulin resistance, type II diabetes, and arterial hypertension [11].

## 2. The Impact of Intermittent Fasting on Lipid Metabolism

Survival and preservation of species continuity depend, amongst others, on their access to food. That is why living organisms have developed many adaptive mechanisms that allow them to survive periods of famine. Some organisms in the periods where they lack access to food are dormant, for example, yeasts entering the stationary phase [18]. In contrast, mammals have liver and adipose tissue. These constitute an energy warehouse for them that allows them to survive during periods of famine [19]. Fats are essential components of the human body. It is a diverse group in terms of the structure and functions fulfilled in the body [20]. One of the most important functions is that of the backup and energy function. Energy is contained within stored adipocytes, which under certain conditions is released from them under the influence of enzymes—lipases [21]. After eating a meal, the concentration of glucose in the body increases and then within a few hours, it returns to the state it was before the meal. The concentration of ketones is low, because glycogen stores in the liver are not depleted [19].

During use of the IF diet, which consists of introducing fasting periods, there are marked metabolic changes in the body [22]. For example, when using a diet during which all food during the day is consumed in a 6-h nutritional window, the glucose level is elevated during and about 6 h after a meal, but remains low for the remaining 16 h until the next day. During the 6–8 h in an 18-h fasting window, ketones remain increased [19]. The human body is naturally adapted to such periods of fasting and in the moment of starving, adaptation mechanisms are used to obtain energy. During fasting, when glucose is exhausted, the body begins to utilize ketones that arise as a result of fatty acid transformations [23,24]. Fatty acids and ketones become the main source of energy for cells. This transition is called intermittent metabolic switching (IMS) or glucose-ketone (G-to-K) switchover. Inverse switching, i.e., ketone-glucose (K-to-G), occurs after the interruption of fasting and meal intake [22].

While the body is abstaining from food, the concentration of glucose, which is the basic energy substrate, decreases. Glycolysis is inhibited. Glycogen reserves in the liver are consumed and the process of gluconeogenesis is activated, during which fats are consumed. In addition, insulin and IGF-1 (insulin-like growth factor-1) levels are reduced in blood and glucagon levels rise. Fatty acids released from fat cells in the process of lipolysis of triacylglycerol and diacylglycerol are released [23]. They are then transported to the liver cells, where they are converted into β-hydroxybutyrate (BHB) and acetoacetate (AcAc) in the β-oxidation process and are further released into the blood and used as a source of energy for body cells, including the brain [24]. Such biochemical changes are accompanied by cellular and molecular adaptations of neuronal networks in the brain. The result is an improvement of their functionality and resistance to stress, injuries, and diseases [23].

The above biochemical transformations of lipids, along with following the IF diet, result in weight loss and changes in lipid parameters. According to studies conducted by Surabhi Bhutani et al., during the use of alternative days on an empty stomach—ADF (alternate day fasting)—for 2–3 weeks showed a reduction in body weight by 3%, while longer attempts to use ADF showed a reduction of 8% and reduced fat mass in viscera. In addition, the levels of total cholesterol (TC) triglycerides and low density cholesterol (LDL) and the size of these molecules were reduced. Changes in these parameters limit the risk of developing coronary heart disease (CHD) [25].

The effects of the IF diet on body weight and LDL cholesterol levels have also been proven in studies conducted by Wilson et al., on 8-week-old mice (39 males and 49 females). These mice were fed high-fat and sugar foods for 24 weeks, and after 12 weeks, they were divided into five groups. The first group were overweight control mice—OBC; the second group were mice without intervention—CON; the third group were mice subjected to the IF diet; the next group were mice subjected to high intensity interval training (HIIT); and the last group were mice subjected to a combination of IF and HIIT. Both IF, and IF and HIIT caused a decrease in body weight and low density lipoproteins (LDL), compared to the HIT and CON groups. These results demonstrate the effectiveness of weight loss despite the simultaneous intake of high-fat and sugar foods [26].

Studies on human individuals are summarized in Table 2.

## 3. The Impact of Intermittent Fasting on Inflammatory Biomarkers

Atherosclerosis is the leading cause of vascular disease in the world. It is a serious problem of pathogenicity and mortality in both developed and developing countries. It is manifested by clinical symptoms, such as ischemic heart disease, peripheral artery disease, and ischemic stroke. It is responsible for acute myocardial infarction and cerebrovascular events, and it is responsible for the most deaths from cardiovascular causes in the world [33,34].

Atherosclerosis is a chronic inflammatory disease during which atherosclerotic plaque form in arterial vessels, which causes sclerosis of the walls and narrowing of the arteries. The development of atherosclerotic plaque is caused by vascular endothelial dysfunction and long-term exposure to cardiovascular disease development factors. One of the most important risk factors is high levels of low density lipoproteins (LDLs) [34]. Excess LDLs accumulated in the sub-epithelial layer of arterial walls is oxidized to oxLDL [35]. This induces an inflammatory response and adhesion to the endothelium of blood leukocytes, mainly monocytes. They migrate to the inner membrane of the vessels and are converted into macrophages [36]. Macrophages, through internalization with oxLDL, are transformed into foam cells that present antigens to immune cells. Activated cells release factors that contribute to smooth muscle cell migration from the medial to the inner membrane [37]. Vascular smooth muscle cells over proliferate and secrete extracellular matrix proteins. There is a further accumulation of lipids both within cells and extracellularly [38]. The majority of risk factors for cardiovascular diseases and factors of atherosclerosis may be modified [39]. One of the modifications is the use of the IF diet.

Inflammation is an important element of development. Pro-inflammatory factors, such as homocysteine, interleukin 6 (IL6), or C reactive protein (CRP), contribute to the development of atherosclerotic plaque. In research conducted by Aksungar et al., the effect of the IF diet on reducing the concentration of the above-mentioned pro-inflammatory factors was demonstrated. The experiment was attended by 40 healthy participants with the correct body mass index (BMI) who fasted during Ramadan and 28 participants, respectively, selected in terms of age and BMI index who did not fast. Venous blood samples to examine the concentration of the above-mentioned pro-inflammatory factors were collected one week before the start of Ramadan, in the last week of fasting, and three weeks after [40].

Adiponectin is a collagen-like plasma protein whose concentration decreases in the course of atherosclerosis, insulin resistance, diabetes, and coronary disease [41]. The use of the IF diet increases the secretion of adiponectin from adipocytes [42]. There is an inverse correlation between plasma adiponectin levels and body weight. Cambuli et al. examined 104 children with obesity. They compared the starting concentration of adiponectin with the concentration observed after one year of a diet and increased physical activity. This concentration increased by 245%. The increase in the adiponectin concentration was proportional to the reduction of body weight [43]. Adiponectin fulfills its functions by acting on adiponectin receptors found in two isoforms—AdipoR1 and AdipoR [44]. It exhibits anti-atherosclerotic and anti-inflammatory effects by inhibiting the adhesion of monocytes to endothelial cells. It also inhibits the excretion of the vascular cell adhesion molecule 1 (VCAM-1), endothelial-leukocyte adhesion molecule 1 (ELAM-1), and intracellular adhesive molecule 1 (ICAM-1) on vascular endothelial cells. This was proven by Ouchi et al. in in vitro studies on human aortic endothelial cells incubated for 18 h in the presence of adiponectin. Adhesion, induced by tumor necrosis factor alpha (TNF-alpha), of THP-1 line monocytes to human aortic endothelial cells was assessed by an adhesion assay. Expression of the molecules was measured by ELISA (enzyme-linked immunosorbent assay). [45,46]. Anti-atherosclerotic action of adiponectin has been proven in many animal models and cell cultures [44]. For example, in studies conducted by Okamoto et al., using reverse transcriptase polymerase chain reaction (real-time) and ELISA testing, it was demonstrated that adiponectin has anti-inflammatory activity in human macrophages by inhibiting the production of CXC 3 receptor chemokine ligands. In in vivo studies on mice deficient in apolipoprotein E/adiponectin, there was an increase in IP-10 in plasma, and increased accumulation of T lymphocytes in vessels and atherosclerosis compared to a single apoE deficiency [47]. Matsuda et al. demonstrated in adiponectin-deficient mice that a deficiency of this protein causes intimal thickening and increases the proliferation and migration of smooth muscle cells by increasing the expression of HB-EGF (heparin-binding epidermal growth factor) [48]. The association of the IF diet with increased concentrations of adiponectin was proven in studies conducted by Wan et al. These studies were carried out on rats assigned to groups with an ad lib diet and with IF for 3 months. Animals with an IF diet were deprived of food for 24 h, every other day. To induce myocardial infarction, the rats’ left coronary artery was ligated. Animals with an IF diet had a higher adiponectin concentration and the area of ischemia was smaller. Moreover, significantly lower inflammatory indexes were observed, leukocytes and IL6, compared to rats with an ad lib diet [49].

An important hormone secreted by adipocytes is leptin [42]. It has a pro-atherogenic effect. Its concentration is elevated in obese people, and is correlated with body mass index (BMI), total cholesterol, triglycerides, blood pressure, and inflammation markers. These correlations were confirmed in studies conducted by Sattar et al., in which leptin concentrations were determined in 550 men with fatal coronary heart disease (fatal CHD) or nonfatal myocardial infarction (nonfatal MI) and in 1184 control patients included in a prospective study on 5561 British men [50]. The concentration of leptin decreases body weight when using the IF diet. Leptin hyperactivity reduces the risk of atherosclerosis by reducing platelet aggregation and decreasing endothelial cell proliferation and migration [51].

Resistin plays an important role in the pathogenesis of atherosclerosis. This is a cytokine derived from adipocytes [52]. Its concentration correlates with resistance to insulin and obesity. It has pro-inflammatory activity [53]. It also promotes the pro-inflammatory activity of neutrophils and macrophages as well as the formation of extracellular deposits in vessels. This happens through the inhibition of AMP-activated protein kinase activation, responsible for the inhibition of neutrophil activity [54]. Resistin increases the expression of chemotactic monocyte 1 protein (MCP-1) and sICAM-1 in vascular endothelial cells. These observations were made by Burnett and his team in studies in which they incubated mouse aortic endothelial cells with a recombinant resistin [52].

Research carried out by Bhutani et al. is proof that the ADF diet shows activity in modulating adipokines. As a result, it has cardio-protective and anti-sclerotic effects. The study included 16 obese people—12 women and 4 men. It lasted for 10 weeks and included three phases of dietary interventions. The first two weeks were the control phase, the next 4 weeks included the ADF diet, in which the feeding time was monitored, and the last 4 week were ADF with a self-fed nutrition time by the patient. After 8 weeks of using the ADF diet, there was a decrease in leptin concentrations, which was associated with a decreased body weight and fat content. The concentration of resistin significantly decreased after using the ADF diet, which probably was associated with a decrease in body weight [42].

Studies on human individuals are summarized in Table 3.

## 4. The Impact of Intermittent Fasting on Blood Pressure

Hypertension is a common disorder of the modern world. In the United States, this problem affects 86 million adults. It is a risk factor for cardiovascular disease, stroke, and chronic kidney disease [57]. It is defined as the occurrence of systolic blood pressure (SBP) in the amount of 140 mmHg and more, or diastolic blood pressure (DBP) of 90 mmHg or more [58].

The use of an IF diet has a beneficial effect on lowering blood pressure. This has been documented in animal studies, and later, the effectiveness of the diet was confirmed in humans. Studies conducted at the University at Buffalo in the United States in male Sprague-Dawley rats confirmed the beneficial effect of the diet on the cardiovascular system. The animals were subjected to a reduced calorie diet or the IF diet was maintained, in which they were fed every other day under a circadian rhythm. To control the heart function, telemetry transmitters were implanted. After a few weeks of observation, a decrease in SBP and DBP blood pressure was noted, as well as a reduction in heart rate [59]. The effectiveness of the diet has also been confirmed in humans in studies conducted at the Buchinger Wilhelmi clinic in Germany. The study group consisted of 1422 people who were subjected to one-year follow-up during the IF diet [22]. The period of fasting was from 4–21 days. Daily meals of 200–250 kcal were accepted. The observed effect of the IF diet on the cardiovascular system was the same as in animals. The study proved the reduction of SBP and DBP in groups of people who fasted for a long period of time. The mechanism of the pressure drop may be associated with an increase in parasympathetic activity due to the brain-derived neurotrophic factor (BDNF), increased norepinephrine excretion through the kidneys, and increased sensitivity of natriuretic peptides and insulin [22]. It was observed that cardiovascular health benefits do not last longer than the period of the IF diet. After its completion, the pressure values return to their initial values [59].

The mechanism of low blood pressure associated with the activation of the parasympathetic system is based on the increased activity of the cholinergic neurons of the cerebrospinal stem [19,60]. Brain-derived neurotrophic factor (BDNF) is mainly produced in response to the activation of glutamatergic receptors, but the IF diet is also somewhat stimulating. The influence of the factor on heart rate and blood pressure has been proven in studies conducted in mice at the George Washington University. Male heterozygous and congenic wild-type mouse mice were tested. In both groups, transmitters were implanted to monitor heart rate. Wild-type mice were infused with recombinant human BDNF into the cerebral ventricles, while mice were infused with the mutated solution of PBS. After 4 weeks of observation, it was noted that the heart rate in mice with intraventricular infusion of the factor was significantly lower compared to mice with PBS infusion. In addition, the changes were independent to the time of day. To explain the mechanism of the reduction of the heart rate in the presence of BDNF, further tests in mice were performed. Both groups were given anti-sympathetic drugs, atenolol, and anti-parasympathetic drugs, atropine. All mice responded to atenolol with a reduction in heart rate. However, with atropine, the heart rate significantly increased in wild-type mice compared to the mutant mice. This study proved the effect of the BDNF factor on the increase in the activity of the parasympathetic system [60]. BDNF increases the synthesis and release of acetylcholine by cholinergic neurons [61]. The cardiac function is controlled by the release of acetylcholine through the vagus nerve to the sinoatrial node, where it reduces the heart rate. In addition, the neurotransmitter expands the blood vessels, causing a reduction blood pressure [62].

Studies on human individuals are summarized in Table 4.

## 5. The Impact of IF Diets on Other Important Factors in the Development of Cardiovascular Diseases

Other modifiable factors of cardiovascular diseases affecting the intermittent fasting diet are, among others, obesity and diabetes. Obesity and being overweight is one of the most predisposing factors for the development of cardiovascular disease, as well as other metabolic syndromes, including diabetes [64,65]. Most evidence supporting the connection between obesity, overweight, and cardiovascular disease (CVD) incidences are based one-time-point measurements. It must be mentioned that prediction will be different for individuals who have just become overweight compared to those who have been obese for years. It was proven in the Framingham Cohort Study that all cause death risk increased simultaneously with an increased duration of obesity (every 2 years lived with obesity, risk of CVD mortality raised 7%). In the studies conducted on humans and on animal models, the beneficial effect of the IF diet on body weight and glycemic parameters was proven. In 2017 Trepanowski et al. provided a single-center randomized clinical trial of obese individuals (mean BMI = 34) to compare the effects of ADF to daily calorie restriction on weight reduction, weight maintenance, and CVD risk predictors [66]. Scientists divided 100 participants into three groups for 1 year (1st group = ADF, 25% energy needs on fast days, 125% of energy needs on feast days; 2nd group = calorie restriction, 75% of energy needs every day; 3rd group = no intervention group). The trial included two phases: 6-month weight loss and 6-month weight maintenance. The authors found that the mean weight loss was similar for the ADF-group and those in the CR-group (calorie restriction; 75% of energy needs every day) at 6 and 12 months. Additionally, it was observed that participants in the ADF group ate more than was prescribed on fast days, and less than prescribed on feast days, while the CR-group followed their prescribed goals without disturbances. It clearly shows that the ADF diet is harder to comply with when compared to the CR system, especially in long term use. Moreover, there were no significant differences between the ADF and CR groups in terms of heart rate, blood pressure, fasting glucose, fasting insulin, triglycerides, and CRP at both time-point assessments [66]. However, the study did not match food intake and meal frequency also did not control for whether participants followed a diet regimen.

In 2018, Schubel et al. conducted a randomized controlled trial to test if the 5:2 diet (ICR—intermittent calorie restriction) is more beneficial in the context of adipose tissue gene expression, anthropometric, body composition, and circulating biomarkers measurements than continuous calorie restriction (CCR) [67]. Investigators randomly assigned 150 overweight and obese nonsmokers to the ICR/5:2 group (5 days without energy restriction and 2 days with 75% energy deficit) and the CCR group (daily energy deficit 20%), or a non-intervention group (control group—without energy restriction). The trial was divided into a 12 weeks intervention phase, 12 weeks maintenance phase, and 26 weeks follow-up phase. Weight change over the intervention phase was −7.1% ± 0.7% (mean ± SEM) with ICR, −5.2% ± 0.6% with CCR, and −3.3% ± 0.6% with the control regimen (*p*_overall_ < 0.001, *p*_ICR vs. CCR_ = 0.053). At the final time-point of week 50, weight loss was −5.2% ± 1.2% with ICR, −4.9% ± 1.1% with CCR, and −1.7% ± 0.8% with the control regimen (*p*_overall_ = 0.01, *p*_ICR vs. CCR_ = 0.89). The authors did not observe any significant differences between the 5:2 diet and the continuous calorie restriction diet in the context of circulating biomarkers and adipose tissue genes [67].

Sutton et al. tried to answer the question of whether health benefits depend on weight loss or other non-weight loss mechanisms in humans. Scientists prepared a proof-concept-study based on early time-restricted feeding (eTRF), a form of intermittent fasting that includes eating early in the day according to the circadian rhythm. Individuals with prediabetes were randomly assigned to eTRF (6 h feeding time, dinner before 3 p.m.) or a control group (12 h feeding time). After 5 weeks of observations, it was proven that eTRF improved beta-cell responsiveness, insulin sensitivity, blood pressure, oxidative stress, and appetite [68].

At the State University of New Jersey, studies were carried out on male C57/BL6 mice in which the effects of the IF diet on the body were studied. Initially, all animals were fed a high-fat diet (45% fat) over a period of 8 weeks to obtain an obese phenotype. The mice were then divided into four groups: The control group included mice on the high-fat ad lib diet (group 1); exercise groups: High-fat diet for two days, then five-day fasting (group 2); low-fat diet (10% fat/group 3); and a group with IF (group 4). After 4 weeks of diet use, a decrease in body weight and body fat content was observed in all experimental groups compared to the control group. Glucose concentration after oral glucose load as well as insulin tolerance were also investigated. The results in all study groups showed lower blood glucose, glycated hemoglobin levels, and increased insulin sensitivity compared to the control group [69]. The effects of the IF diet on weight loss were also confirmed in studies in the human population in Manchester. Women who were overweight or obese in the pre-menopausal age were subjected to an attempt to reduce calories by 25% based on the Mediterranean diet for 6 months. After the end of the study, a decrease in weight was observed in women along with a reduction of abdominal circumference and a decrease in adipose tissue content. Reductions in weight also had a positive effect on well-being. In addition to body weight, women were also tested for insulin sensitivity and glucose. The results also proved the beneficial effect of the IF diet on glycemic parameters. Insulin resistance in the study group was assessed using the HOMA index [27].

To determine the effect of the IF diet on glucose metabolism, the study also investigated people with diabetes. The particularly positive effects of IF diets were observed in patients with type 2 diabetes. Type 2 diabetes is a common metabolic disorder in the world. It correlates with an increase in obesity rates and sedentary lifestyles. Limiting the development of diabetes prevents many diseases, including cardiovascular diseases, neuropathy, retinopathy, and kidney disease [70]. Diabetes induced by obesity is characterized by hyperglycemia, insulin resistance, and progressive beta cell failure. The use of the IF diet effectively improves glucose metabolism in patients with type 2 diabetes. This was demonstrated in the Diabetes Remission Clinical Trial (DiRECT) in which diabetes mellitus after a hypocaloric diet was observed. Participants under constant medical care consumed meals of about 850 calories/day for 12 weeks [71]. It was proven that weight loss normalizes fasting blood glucose, significantly reduces glycated hemoglobin (HBa1c), and increases insulin sensitivity in people with type 2 diabetes [71,72]. The mechanism of this phenomenon is associated with increased sensitivity of the insulin receptor after the IF diet, due to which insulin stimulates quick uptake of glucose by muscle cells and hepatocytes [73]. However, the return to daily nutritional conditions resulted in fasting glucose concentrations returning to the baseline values [70].

There is also evidence of an effect of the IF diet on the activity of pancreatic B-cells in patients with type 1 diabetes. This is confirmed by the study on rats with streptozotocin-induced diabetes. At the same time, the control group was tested and the citrate buffer was injected. For 30 days, the animals were exposed to starvation during the night or had a limited food supply. After observation, a decrease in glucose concentration, an increase in insulin in plasma, a decreased HOMA index, and an increase in the number of pancreatic B-cell cells in streptozotocin-induced rats on the IF diet [74] were observed. Decreased apoptosis of B cells under the influence of diet was also observed in mice with type 2 diabetes [75]. There is not much known about the mechanisms by which diet improves glucose metabolism. There is a hypothesis that the use of the IF diet does not increase insulin sensitivity, but increases the mass of pancreatic islet B cells, which in turn causes an increase in insulin in the plasma, reducing blood glucose. Another hypothesis is the impact of autophagy on beneficial effects of the IF diet in diabetic patients. In the conditions of the IF diet, an increased activity of autophagy in the pancreatic islets is observed, which leads to an improvement in glucose tolerance as a result of increased insulin secretion. The study also observed an increase in the expression of the Ngn3 pancreatic regeneration marker, which plays a key role in the maturation of pancreatic B-cells [76]. Although the mechanisms of the influence of the IF diet on diabetes remedies are not fully understood, its beneficial effects can be applied as a potential therapy for treatment.

In addition to obesity and diabetes, a risk factor for cardiovascular disease is also cerebrovascular diseases, including stroke. Stroke is the necrosis or damage to brain areas as a result of interrupted blood supply. There are two subtypes of stroke: Ischemic stroke and hemorrhagic stroke. This condition causes a wide range of disability depending on the extent of the stroke [77]. The IF diet has also had a beneficial effect on the prevention of stroke. This was confirmed in studies at the National University of Singapore conducted on young mice (3 months) and middle aged mice (9 months). The animals were subjected to an experimental focal ischemic stroke and then subjected to an IF diet. After observation, it was concluded that the use of the IF diet reduces the risk of stroke and its extent. The mechanism of this phenomenon is related to the protective action of the brain tissue against oxidative stress, and it was proven that the diet regulates many neuroprotective proteins present in the mouse brain after stroke. These proteins include BDNF, the basic fibroblast growth factor (bFGF), stress response protein (Hsp70), and glucose-regulated protein 78 (GRP78). It is believed that the protective effect of the IF diet on stroke is associated with the activation of adenosine monophosphate-activated protein (AMPK) and SIRT1 protein in response to reduced energy during fasting. AMPK and SIRT1 regulate neuroprotective proteins, preventing pathological processes in brain cells [78].

Cardiovascular disease development is also predisposed with cardiac hypertrophy. Wang et al. determined the effect of a long-term high-fat diet (HFD) and an IF diet on the hearts of mice. After 11 months, the cross-section of cardiomyocytes in HFD-fed mice was increased, whereas the IF diet did not induce cardiac myopathy in mice. The intense fasting increased the active caspase 3, suggesting that intermittent hunger may increase apoptosis and reduce autophagy in the heart. The mechanism of the influence of the IF diet on normal heart mass, however, has not been thoroughly studied [79].

Studies on human individuals are summarized in Table 5.

## 6. Importance of Food Quality in the “Eating Window”

Diet plays an important role in the prophylaxis of cardiovascular diseases. Special attention should be paid to nutraceuticals, which contain many beneficial substances for the human organism. These substances, to name a few, are polyphenols, resveratrol, carotenoid, polyunsaturated fatty acids (PUFAs), curcumin, and zinc [80]. Carotenoids are one of the basic ingredients in the Mediterranean diet. They are present in vegetables, (especially in carrots), fruits, and also in seaweed. Their beneficial effect in preventing cardiovascular events is not yet fully known. However, they are attributed to antioxidant and anti-inflammatory functions due to the influence on lipoxygenases [81]. A nutraceutical deserving specific consideration is resveratrol. Its biologically active isomer is trans 3,5,4′-trihydroxystilbene. Grapes are rich in resveratrol, which is why the largest concentration is found in red wine, but it is also found in blueberries, peanuts, and pistachios [80]. It has antioxidant properties and it is helpful in the treatment of many disorders due to its cardioprotective effect. It was observed that resveratrol may improve blood pressure. Wiciński et al. showed that a dose of 10 mg/kg of resveratrol per day increases the concentration of BDNF and reduces vascular smooth muscle cells contractility [82]. Moreover, the same author proved that resveratrol (10 mg/kg per day) may increase adiponectin concentrations, but the exact mechanism has not fully been elucidated [83].

Resveratrol inhibits hypercholesterolemia development. This was confirmed in tests conducted on mice by Chen et al., who observed a decrease in lipid parameters within 8 weeks of a high fat diet and administration of resveratrol in the dose of 200 mg/kg per day. Increased lipid metabolism after administration of resveratrol is associated with elevated 7-α-hydroxylase activity in mice. The enzyme is regulated by the liver receptor, LXR. It is involved in the cholesterol to 7-hydroxycholesterol transformation and then to cholic acid, which results in increased production of bile acids and lowers cholesterol in hepatocytes [84]. Nonetheless, in the analysis done by Sahebkar on human models, no beneficial effects of resveratrol on dyslipidemia were observed. This can be correlated with the metabolism of resveratrol as well as the first passing through the liver, which results in its decrease in activity in the blood [85].

Polyphenols also contribute to the reduction of the lipid profile. Their highest concentration can be found in mulberry leaves. The role of polyphenols in the reduction of lipid concentration is attributed to the inhibition of the activity of the enzymes responsible for their synthesis. These include fatty acid synthetase, 3-hydroxy-3-methylglutarylCoA reductase, or acetyl-CoA carboxylase. Therefore, polyphenol extract from mulberry leaves decreases the culmination of fatty acids in the liver through the activation of the AMP protein kinase pathway [86]. Other polyphenols, which also diminish the lipid profile, are found in black tea theaflavin. This has been documented in research overseen by Jin et al. on rat models with a high-fat diet. The rats were fed black tea extract containing highly purified mixtures of theaflavins at the same time. After the completion of the research, it was observed that the total cholesterol concentration, LDL-C, and triglycerides were slightly decreased. Theaflavin reduced the atherogenicity index, in addition to also ceasing alanine transaminase and hepatic lipase activity [87]. Advantageous outcomes of polyphenol were also proven in the research of Gunathilake, Wang, and Vasantha Rupasinghea. The study subjected rats with hypertension, which were fed with the AIN-93G diet as the control group, the second group was fed a high fat diet, while the third group was fed different doses of polyphenol rich fruit juices. The researchers documented that the polyphenol rich juice supplement successfully lowered the total cholesterol values and LDL-C in serum in addition to the cholesterol concentration in the liver. Apart from the hypolipidemic function, blood pressure reduction was also recognized [88].

Protective actions in cardiovascular diseases also have unsaturated fatty acids, mainly omega-3 fatty acids: Eicosapentaenoic acid (EPA) and docosahexaenoic acid (DHA). A rich source of these ingredients is fish oil [89]. Supplementation of unsaturated fatty acids reduces the concentration of triacylglycerol from 25% to 30%, but can increase the concentration of LDL cholesterol in serum [90]. Schmidt et al. observed a decrease in triglyceride levels in dyslipidemia patients receiving polyunsaturated n-3 fatty acids (PUFA). This was connected with the overexpression of peroxisome proliferator-activated receptor (PPAR). Additionally, this diet reduces the triacylglycerol levels as the result of decreased gene expression responsible for triacylglycerols’ synthesis (MOGAT3, MOGAT2, and DGAT1). Such a diet also increases lipase lipoprotein (LP) activity and the VLDL catabolism. This influences the inhibition of the expression of apoCIII and apoB, and through this, lowers VLDL production [91].

It is also worth including curcumin and zinc in the diet as they have proven anti-atherosclerotic action. According to the studies by Zhao et al., conducted on mice with apolipoprotein E deficiency, treatment with curcumin inhibits macrophage transformation in foam cells. Moreover, they also observed that curcumin therapy decreases oxLDL accumulation in macrophages. Curcumin suppresses scavenger receptor class A (SR-A) expression and induces ATP-binding cassette A1 (ABCA1) synthesis. This is followed by prevention of oxLDL binding to SR-A and increased outflow of ABCA1-dependent cholesterol [92].

Rahimi-Ardabili et al. showed that in hemodialyzed patients, administration of zinc may induce a higher activity of paraoxonase enzymes [93]. These enzymes are located on cholesterol HDL molecules, which prevent LDL oxidation [94].

## 7. Advantages and Disadvantages of Using the IF Diet

There are many studies conducted on humans and animals confirming the therapeutic effectiveness of the IF diet [11]. It reduces body fat and body mass, which supports the healthy functioning of the cardiovascular system, and reduces the incidence of myocardial infarction [95]. Individuals can influence the concentration of many metabolic biomarkers, for example, the concentration of insulin and glucose, thereby reducing the risk of metabolic syndrome [13]. It also reduces the risk of type 2 diabetes [9]. There are studies confirming the impact of long-term use of the IF diet on the extension of the viability of individuals [14]. The IF diet positively affects the functioning of the nervous system. By affecting the reduction of free radical formation in the body and stress response systems, it protects neurons from environmental and genetic factors that cause them to age [95].

Intermittent fasting also has its drawbacks. Periods of fasting of a few hours at the start cause huge problems. This is accompanied by a bad mood at the beginning of the diet, such as fatigue or dizziness, because the body needs time to get used to using ketones instead of glucose. Certainly, this is not a good diet for patients with reactive hypoglycemia. Moreover, caloric restriction with the simultaneous use of antidiabetic drugs may lead to severe hypoglycemia and even death [96]. In the elderly, it is associated with an increased risk of cardiovascular disease, arrhythmia, and stroke. Fluctuations in glucose concentration cause instability of the body, which results in an increased number of falls and frequent fractures due to osteoporosis [97]. The ACCORD trial confirmed greater risk of cardiovascular events during the presence of hypoglycemia in both older and younger individuals [98]. Higher risk of diabetic ketoacidosis is also not without significance, especially when there is not enough insulin due to low food intake during fasting.

In addition, excessive restriction of calories causes dysregulation of hormone management. Such disturbances may cause menstrual cycle disorders in women and reduced testosterone in men. Intermittent fasting should not be used by children, pregnant women, and people performing heavy physical work [99].

## 8. Summary

The IF diet limits many risk factors for the development of cardiovascular diseases and therefore the occurrence of these diseases. Fatty acids and ketones become the main energy fuel, because the body undergoes metabolic switching of glucose-ketone (G-to-K). By affecting the biochemical transformations of lipids, it decreases body mass and has a positive influence on lipid profile parameters—it reduces the concentration of total cholesterol, triglycerides, and LDL cholesterol.

Benefit from the use of the IF diet were confirmed in research on the development of atherosclerosis. Intermittent fasting inhibits the development of atherosclerotic plaque by reducing the concentration of inflammatory markers, such as IL-6, homocysteine, and CRP. The IF diet results in an increase in plasma concentrations of adiponectin and a decrease in leptin and resistin concentrations. By altering the levels of these adipokines, it inhibits the adhesion of monocytes to vascular endothelial cells, neutrophils, and macrophage pro-active activity, and platelet aggregation. The transformation of macrophages into foam cells, the formation of extracellular deposits in vessels, and the proliferation and migration of endothelial cells into the inner arterial vascular membrane are limited.

The beneficial effect of the diet was observed in the prevention of hypertension. The intermittent fasting diet causes an increase of BDNF factor, which results in lowering the systolic and diastolic blood pressure by activating the parasympathetic system. BDNF causes acetylcholine to be released by the vagus nerve, which reduces the frequency of heart contractions.

The positive effect of the IF diet has also been documented in obese and diabetic people. The reduced amount of food consumed when using the IF diet results in a decrease in body weight. It also improves glucose metabolism and increases the sensitivity of tissues to insulin by increasing the B cells of the pancreatic islets. The IF diet also limits cardiac hypertrophy.

It remains questionable if these benefits are solely due to weight loss or non-weight loss mechanisms. The success of every type of diet depends on rule compliance—following a prescribed diet according to the circadian rhythm.

Despite the intermittent fasting diet having many benefits, its disadvantages are not without significance. Fasting may be dangerous and it is not recommended for people with hormonal imbalances, pregnant and breastfeeding women, and diabetics. Moreover, people with eating disorders, a BMI under 18.5, and underweight people are also not recommended to use the intermittent fasting diet.

In recent years, the IF diet and its varieties have become increasingly popular. This diet not only serves to reduce body weight, but can also be used as an effective non-pharmacological treatment method. This has been proven through various studies performed on people and animals. However, individuals’ current health and situation should be considered before commencing the IF diet.

## Figures and Tables

**Table 1:** Comparison of intermittent fasting protocols [8,11].

Energy restriction for two nonconsecutive days and ad libidum intake for other five days.	Eating days—ad libidum food intake. Fasting days—Low or no-energy food intake.	Ad libitum food intake in specific timeframe (~12-h). Night fasting period according to circadian rhythm.	
**5:2 Protocol**	**ADF Protocol**	**TRF Protocol**	**Days of the Week**
Fast	Eat	~12-h	Monday
Fast	Fast	~12-h	Tuesday
Eat	Eat	~12-h	Wednesday
Eat	Fast	~12-h	Thursday
Eat	Eat	~12-h	Friday
Fast	Fast	~12-h	Saturday
Fast	Eat	~12-h	Sunday

Abbreviations: ADF, alternate-day fasting; TRF, time-restricted feeding.

**Table 2:** Impact of intermittent fasting on lipid profile.

First Author and Reference Number	Number of Enrolled	Participants Description	Time	Lipids	NCT Number
Harvie et al., 2011 [27]	107	Overweight or obese premenopausal women	6 months	NS (LDL, TGs, HDL)	NCT02679989
Varady et al., 2013 [28]	15	Overweight individuals BMI 20–29.9 kg/m^2^	12 weeks	↓TC (*p* < 0.01) ↓LDL (*p* < 0.01) NS HDL ↓TGs (*p* < 0.01)	NCT00960505
Bhutani et al., 2013 [25]	83	Obese individuals BMI 30–39.9 kg/m^2^	12 weeks	NS TC ↓LDL (*p* < 0.05) NS TGs ↑HDL (*p* < 0.05)	NCT00960505
Eshghinia et al., 2013 [29]	15	Overweight or obese women BMI ≥ 25 kg/m^2^	8 weeks	NS (LDL, TGs, HDL)	-
Teng et al., 2013 [30]	28	Malay Men BMI 23–29.9 kg/m^2^	12 weeks	↓TC (*p* < 0.001) ↓LDL (*p* < 0.05) NS HDL NS TGs	NCT01665482
Harvie et al., 2013 [31]	77	Overweight or obese women	3 months	NS (LDL, TGs, HDL)	NCT00869466
Chowdhury et al., 2016 [32]	23	Obese individuals BMI 30–39.9 kg/m^2^	6 weeks	NS (LDL, TGs, HDL) ↑TC	-

Abbreviations: NS, not statistically significant (*p* > 0.05); LDL, low-density lipoprotein; TGs, triglycerides; HDL, high-density lipoprotein; TC, total cholesterol. Only studies from the past 10 years with full data published were considered.

**Table 3:** Impact of intermittent fasting on inflammatory markers’ concentration.

First Author and Reference Number	Number of Enrolled	Participants Description	Time	Inflammatory Biomarkers	NCT Number
Harvie et al., 2013 [31]	77	Overweight or obese women	3 months	NS (IL6, TNFα, leptin, adiponectin)	NCT00869466
Varady et al., 2013 [28]	15	Overweight individuals BMI 20–29.9 kg/m^2^	12 weeks	↓CRP (*p* = 0.01) ↓Leptin (*p* = 0.03) ↑Adiponectin (*p* < 0.01)	NCT00960505
Bhutani et al., 2013 [25]	83	Obese individuals BMI 30–39.9 kg/m^2^	12 weeks	NS CRP	NCT00960505
Hoddy et al., 2016 [55]	59	Obese individuals BMI 30–39.9 kg/m^2^	10 weeks	↓Leptin (*p* < 0.05)	-
Chowdhury et al., 2016 [32]	23	Obese individuals BMI 30–39.9 kg/m^2^	6 weeks	NS (IL6, CRP, leptin, adiponectin)	-
Safavi et al., 2017 [56]	34	Male individuals 16–64 years old (Ramadan)	4 weeks	NS (adiponectin, TNFα)	-

Abbreviations: NS, not statistically significant (*p* > 0.05); IL6, interleukin 6; CRP, C-reactive protein; TNFα, tumor necrosis factor α; Only studies from the past 10 years with full data published were considered.

**Table 4:** Impact of intermittent fasting on blood pressure.

First Author and Reference Number	Number of Enrolled	Participants Description	Time	Blood Pressure	BDNF	NCT Number
Harvie et al., 2011 [27]	107	Overweight or obese premenopausal women	6 months	↓Systolic (*p* = 0.99) ↓Diastolic (*p* = 0.84)	NS	NCT02679989
Varady et al., 2013 [28]	15 (5 M/10 F)	Overweight individuals BMI 20–29.9 kg/m^2^	12 weeks	↓(*p* = 0.51)	-	NCT00960505
Bhutani et al., 2013 [25]	83 (3 M/80 F)	Obese individuals BMI 30–39.9 kg/m^2^	12 weeks	↓Systolic (*p* = 0.254) ↓Diastolic (*p* = 0.570)	-	NCT00960505
Eshghinia et al., 2013 [29]	15 F	Overweight or obese women BMI ≥25 kg/m^2^	8 weeks	↓Systolic (*p* < 0.001) ↓Diastolic (*p* < 0.05)	-	-
Teng et al., 2013 [30]	28 M	Malay Men BMI 23–29.9 kg/m^2^	12 weeks	↓Systolic (*p* < 0.05) ↓Diastolic (*p* < 0.05)	-	NCT01665482
Erdem et al., 2018 [63]	60	Individuals from the Cappadocia cohort with prehypertension and hypertension SBP 12—139 and ≥140; DBP 80–89 and ≥90 mmHg		↓Systolic (*p* < 0.001) ↓Diastolic (*p* < 0.039)	-	

Abbreviations: SBP, systolic blood pressure; DBP, diastolic blood pressure; BDNF, brain-derived neurotrophic factor; M, male; F, female. Only studies from the past 10 years with full data published were considered.

**Table 5:** The impact of intermittent fasting on obesity and glycemic profile.

First Author and Reference Number	Number of Enrolled	Participants Description	Time	Weight Changes	Glycemic Profile	NCT Number
Harvie et al., 2011 [27]	107	Overweight or obese premenopausal women	6 months	NS	↓insulin NS glucose	NCT02679989
Harvie et al., 2013 [31]	77	Overweight or obese women	3 months	NS	↓insulin NS (HbA1C, glucose)	NCT00869466
Varady et al., 2013 [28]	15	Overweight individuals BMI 20–29.9 kg/m^2^	12 weeks	↓(*p* < 0.001)	-	NCT00960505
Bhutani et al., 2013 [25]	83	Obese individuals BMI 30–39.9 kg/m^2^	12 weeks	↓(*p* < 0.05)	NS (insulin, glucose)	NCT00960505
Hoddy et al., 2016 [55]	59	Obese individuals BMI 30–39.9 kg/m^2^	10 weeks	↓(*p* < 0.0001)	↓(insulin, glucose)	-
Chowdhury et al., 2016 [32]	23	Obese individuals BMI 30–39.9 kg/m^2^	6 weeks	↑(NS)	NS (insulin, glucose)	-
Safavi et al., 2017 [56]	34	Male individuals 16–64 years old (Ramadan)	4 weeks	NS	-	-
Trepanowski et al., 2017 [66]	100	Obese individuals BMI 34	12 months	↓(comparing to control group)	↓(insulin, glucose) comparing to control group	NCT00960505
Schubel et al., 2018 [67]	150	Obese and overweight BMI ≥ 25	50 weeks	↓(comparing to control group)	NS (insulin, glucose) comparing to control group	NCT02449148

Abbreviations: NS-non significant. Only studies from the past 10 years with full data published were considered.
